# Exploring the relationship between somatosensory-evoked potentials, resting-state theta power, and acute balance performance

**DOI:** 10.1038/s41598-025-23878-z

**Published:** 2025-10-16

**Authors:** Rouven Kenville, Dennis Groß, Maximilian Helbich, Patrick Ragert, Tom Maudrich

**Affiliations:** 1 Department of Movement Neuroscience, Faculty of Sport Science, University of Leipzig, Leipzig, Germany; 2 Department of Neurology, Max Planck Institute for Human Cognitive and Brain Sciences, Leipzig, Germany

**Keywords:** Acute balance performance, Somatosensory-evoked potentials, Electroencephalography, Slackline, Motor control, Predictive markers

## Abstract

Balance represents a fundamental motor ability whose considerable inter-individual variability and susceptibility to prior experience and task specificity complicate its assessment. Neurophysiological measures such as electroencephalography (EEG) and somatosensory-evoked potentials (SEPs) offer complementary windows into the sensorimotor mechanisms that underpin balance control and may be associated with individual differences in acute performance levels. In the present study, 25 healthy adults naïve to slacklining underwent tibial nerve SEP recordings using single-pulse and paired-pulse paradigms on both dominant and non-dominant legs to assess excitation and inhibition in the sensorimotor cortex. This was followed by five minutes of resting-state EEG. Participants then completed three balance tasks on a slackline: single leg stance with eyes open, single leg stance with eyes closed, and a successive steps task, on each leg. SEP amplitude and paired-pulse inhibition, as well as resting-state theta power were taken as neurophysiological measures. Analysis revealed a correlation between lower resting-state theta power and superior single leg stance performance with eyes-open on the non-dominant leg, while no significant relationships emerged for the eyes-closed or successive steps tasks. Furthermore, neither SEP amplitudes nor paired-pulse inhibition were significantly associated with any balance outcome. Collectively, the present findings indicate that resting-state theta power could be a non-invasive marker of acute balance performance. These results underscore the promise of spectral EEG measures for acute assessment of specific sensorimotor capacity and suggest that future research should explore their utility in clinical rehabilitation and performance monitoring.

## Introduction

Balance is a fundamental motor ability that underpins functional independence across the lifespan and serves as a sensitive marker of neuromuscular aging^1,2^. Due to the considerable inter-individual variability in balance ability^3^, systematic assessment is essential for informing clinical rehabilitation strategies and optimizing performance outcomes^4,5^. However, despite growing interest, current approaches to understanding individual differences in fundamental motor abilities across both healthy and clinical populations remain limited^6,7^. When studying fundamental motor abilities such as balance, it is essential to move beyond familiar tasks, whose outcomes are influenced by prior experience. Investigating novel motor challenges offers a unique opportunity to minimize experience bias, identify markers linked to innate motor aptitude, and inform the development of targeted interventions to facilitate these abilities^4^. Another important consideration is the timing of performance assessment, namely whether to evaluate motor performance acutely, in a single session or longitudinally across multiple sessions^8^. Considering the above, the present study focused on acute balance performance (without prior practice) in an unfamiliar task, to (1) ensure that observed variability reflects specific sensorimotor capacity and (2) to isolate baseline neurophysiological markers associated with specific balance performance. Given its dynamic nature and established relevance in both athletic training^9^ and clinical rehabilitation^10,11^, slacklining was selected as a novel balance paradigm well-suited to probing individual differences in acute balance performance.

Neurophysiological modalities, such as somatosensory evoked potentials (SEP) and resting-state electroencephalography (EEG), provide valuable insights into balance performance by examining the integrity of somatosensory pathways and the dynamics of cortical networks that underlie postural control^12,13^. SEPs represent neural responses to sensory stimuli, reflecting evoked somatosensory processing^12^. Short-latency SEP amplitudes following upper or lower extremity stimulation have been suggested as potential markers of motor performance^14^. While evidence is limited, previous research has shown SEP amplitude modulation in athletes, with some studies reporting use-dependent increase of SEP amplitudes^15,16^. For example, Murakami et al. (2008) stimulated bilateral median and tibial nerves in non-athletes, football players, and racquet players. They found elevated tibial nerve SEP amplitudes in football players and heightened median nerve SEP amplitudes in racquet players, suggesting a link between somatosensory processing and long-term exercise adaptations specific to the trained limbs. Findings remain inconclusive, however, as other studies have failed to observe similar effects^17,18^. Previous research has also shown athletes to demonstrate superior gating compared to non-athletes^19^. Paired-pulse inhibition (PPI), a phenomenon where the amplitude of a second SEP response is reduced when two stimuli are presented in close succession can serve as a marker of sensorimotor gating and serves as a proxy for inhibitory circuits in the sensorimotor cortices^20^. Although the association between PPI and fundamental motor abilities remains unexplored, a previous study has demonstrated its modulatory potential in response to acute exercise^21^.

Studies on resting-state brain activity have provided valuable insights into individual differences in brain function and their relationship to behavior^22^. These resting-state markers are influenced by experience^23^, reflect the organization of brain networks engaged during task performance and have been shown to correlate with subsequent behavioral outcomes^24^. One such marker, spectral power of EEG signals, measures cortical synchronization and has been utilized to predict motor performance^7^. Theta power (4–8 Hz), particularly in fronto-central regions, has been implicated in postural stability^25^. Several EEG studies have demonstrated increased theta power during balance tasks, such as standing on one leg or walking a balance beam^26,27^. The observed increase in theta activity is thought to support error detection and postural monitoring^26^. Crucially, theta power in frontal, central, and parietal regions has been shown to positively correlate with improved balance performance as task difficulty increases^28^, indicating its potential as a neurophysiological marker to assess acute balance performance.

In summary, this study examines whether short-latency SEP amplitudes and resting-state theta-band EEG power are associated with acute balance performance on a slackline. Based on the outlined research, we hypothesize that elevated short-latency SEP amplitudes and greater resting-state theta power will each correlate with superior acute balance performance. Furthermore, it has been shown that increased excitation is typically associated with decreased inhibitory control^29,30^. Hence it is reasonable to assume that the amount of PPI is associated with individual variations in acute balance performance. Therefore, on an exploratory level, we further assume a significant association between reduced PPI and acute balance performance. With this study, we hope to advance the understanding of neurophysiological markers associated with acute balance performance while adding to the development of targeted balance training and rehabilitation strategies.

## Results

### Slackline performance

For the SEP sample (*n* = 20), balance performance did not differ significantly between SL-EO_D_ and SL-EO_ND_ (z = −1.045, *p* = 0.312, r_bs_ = −0.267), nor between SL-EC_D_ and SL-EC_ND_ (z = 0.458, *p* = 0.648, r_bs_ = 0.124). We did, however, observe a significantly higher step-count for STEP_fw_ as compared to STEP_bw_ (z = 2.448, *p* = 0.013, r_bs_ = 0.743). Similarly, for the EEG sample (*n* = 22), no differences were observed between SL-EO_D_ and SL-EO_ND_ (z = −1.153, *p* = 0.262, r_bs_ = −0.281), nor between SL-EC_D_ and SL-EC_ND_ (z = 0.828, *p* = 0.424, r_bs_ = 0.202). Again, we observed a significantly higher step-count for STEP_fw_ as compared to STEP_bw_ (z = 2.794, *p* = 0.005, r_bs_ = 0.848).

### Somatosensory-evoked potentials (SEP)

For an initial comprehensive overview, Tables 1 and 2 display the latencies and amplitudes of all relevant components. Participants received stimulation at 11.45 ± 1.77 mA (mean ± SD) for the dominant leg and 12.48 ± 2.81 mA for the non-dominant leg. Notably, no significant differences in stimulation intensities were observed between the dominant and non-dominant legs, neither for single-pulse stimulation (t = −1.746, *p* = 0.097, d = −0.390), nor for paired-pulse stimulation (t = −1.784, *p* = 0.090, d = −0.399). Furthermore, we did not find significant differences between dominant and non-dominant legs, neither for SEP amplitudes (F(1,19) = 0.245, *p* = 0.626, η_p_^2^ = 0.013), nor for PPI (F(1,19) = 1.051, *p* = 0.318, η_p_^2^ = 0.052).

**Table 1:** Latencies of single-pulse and paired-pulse SEP components for dominant and non-dominant legs (values are expressed in milliseconds (ms) as mean ± SD).

	Single-Pulse				Paired-Pulse			
	N30	P40	N50	P60	N30	P40	N50	P60
Dominant	32.14 ± 3.43	41.50 ± 3.02	51.12 ± 3.48	61.72 ± 3.93	94.41 ± 3.56	102.45 ± 3.26	111.81 ± 3.59	120.51 ± 4.32
Non-Dominant	30.14 ± 3.22	42.36 ± 3.11	51.51 ± 3.41	61.75 ± 3.89	92.68 ± 2.77	102.78 ± 3.36	112.91 ± 3.95	121.65 ± 4.71

**Table 2:** SEP amplitudes and corresponding paired-pulse Inhibition (PPI) of dominant and non-dominant legs (values are expressed in microvolts (µV) as mean ± SD).

			N30-P40	P40-N50	N50-P60
Dominant	Amplitude (SP)		1.86 ± 1.16	2.90 ± 2.33	2.48 ± 1.70
	PPI		0.54 ± 0.35	0.79 ± 0.45	0.64 ± 0.55
Non-Dominant	Amplitude (SP)		2.07 ± 0.79	2.50 ± 1.40	2.24 ± 1.25
	PPI		0.65 ± 0.30	0.89 ± 0.38	0.60 ± 0.38

**Fig. 1:**
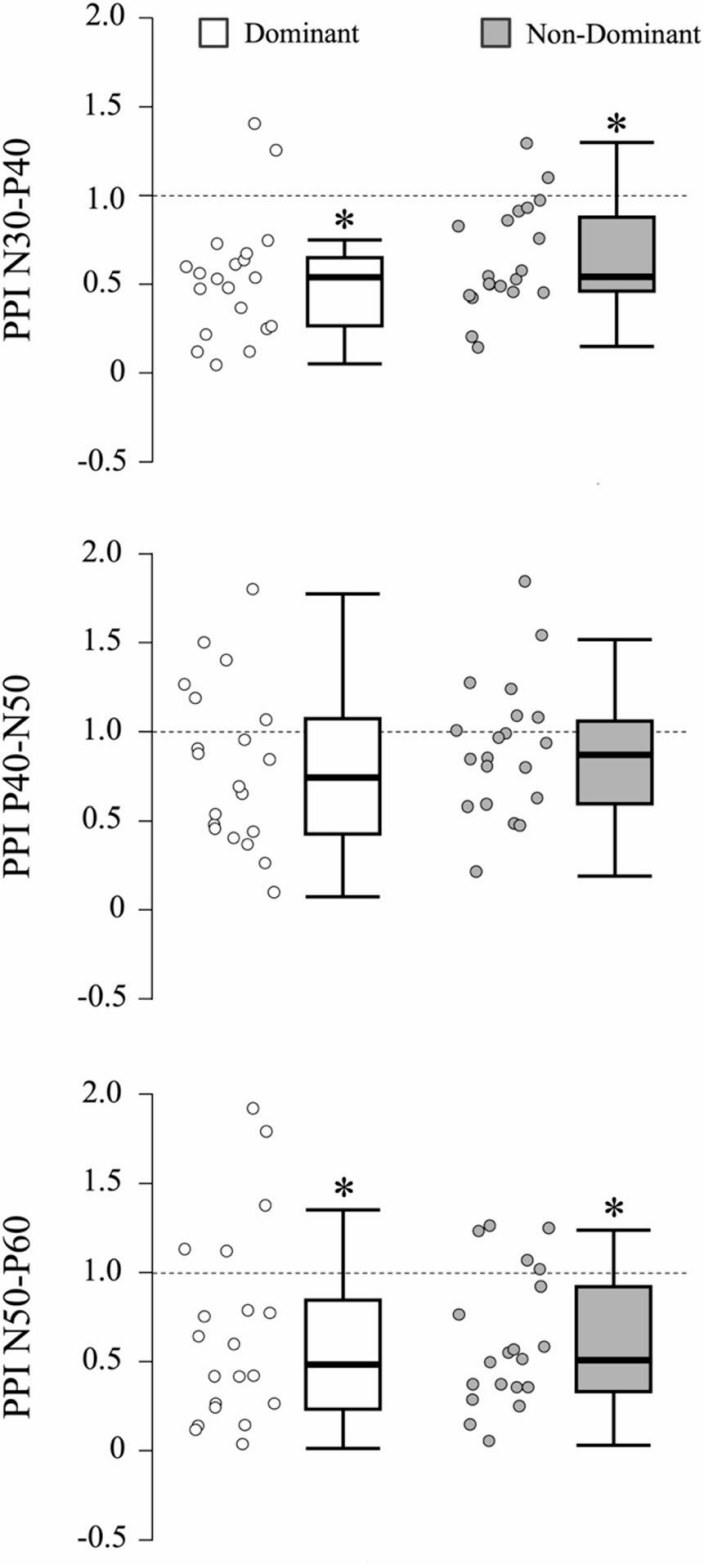
Paired-pulse inhibition. Each row presents paired-pulse inhibition (PPI) for a specific SEP component, with white boxplots representing the dominant leg and gray boxplots the non-dominant leg. A dashed horizontal line denotes the reference value of 1, and asterisks indicate statistically significant differences at α = 0.05.

For the dominant leg, paired-pulse stimulation induced significant inhibition (i.e., PPI < 1) for N30-P40 (MD = −0.463, t = −5.992, *p* < 0.001, d = −1.340) and N50-P60 (MD = −0.356, t = −2.899, *p* = 0.009, d = −0.648), but not for P40-N50 (MD = −0.214, t = −2.109, *p* = 0.048, d = −0.214) after adjusting for multiple comparisons. Similarly, analysis of the non-dominant leg showed significant inhibition for N30-P40 (MD = −0.348, t = −5.232, *p* < 0.001, d = −1.170) and N50-P60 (MD = −0.403, t = −4.726, *p* < 0.001, d = −1.057), but not for P40-N50 (MD = −0.113, t = −1.310, *p* = 0.206, d = −0.293). Detailed PPI results can be found in Table 2 while Fig. 1 provides a comprehensive overview of PPI results.

Spearman correlational analyses did not reveal any significant associations between SEP amplitudes and SL-EC, SL-EO, or STEP for either leg. Similarly, no significant correlations were observed between PPI and SL-EC, SL-EO, or STEP for either leg.

### Relative theta power

**Fig. 2:**
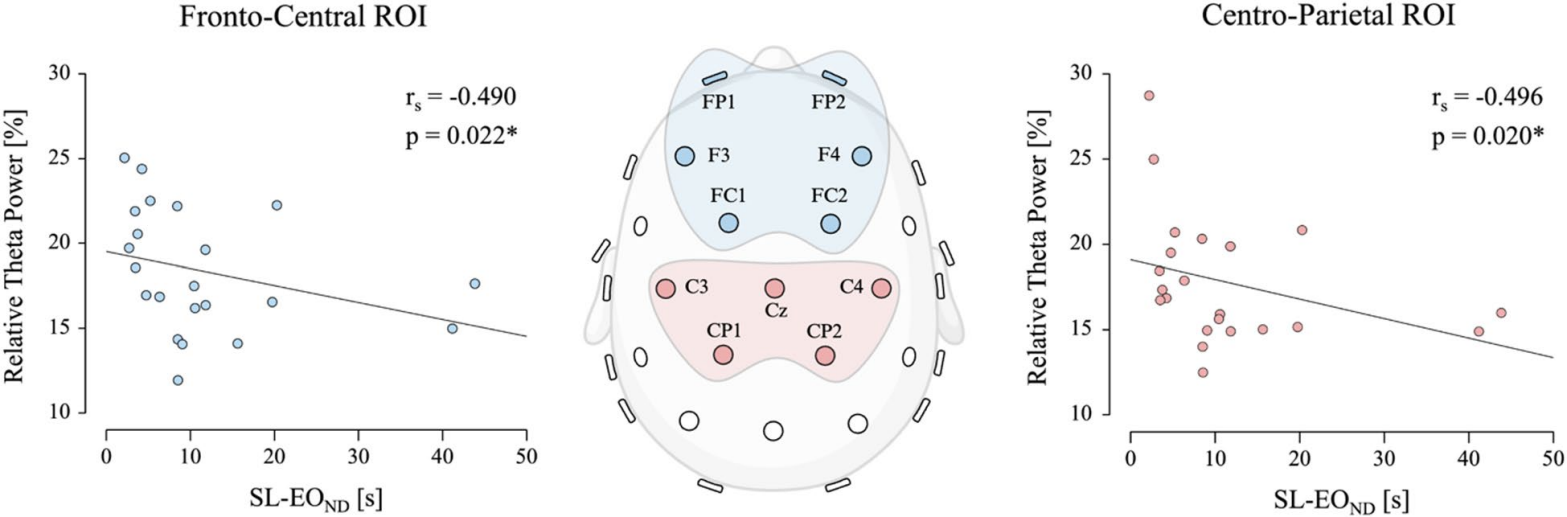
Relative theta power. Scatterplots illustrate the relationship between relative theta power spectral density and single-leg stance with eyes open on the non-dominant leg (SL-EO_ND_). The left panel represents the fronto-central ROI (blue), while the right panel represents the centro-parietal ROI (red). Spearman’s rho and associated p-values are reported within each panel. The central topographic map shows the electrode sites comprising each ROI. Asterisks denote correlations that remain significant after Bonferroni correction at α_bonf_ = 0.025.

We observed a significant negative correlation between SL-EO_ND_ and relative theta power in both fronto-central (r_s_ = −0.490, *p* = 0.022, 95% CI [−0.755 −0.086]) and centro-parietal ROI’s (r_s_ = −0.496, *p* = 0.020, 95% CI [−0.759 −0.094]) (Fig. 2). Although similar in direction, the same relationship was not observed between SL-EO_D_ and relative theta power, neither in fronto-central (r_s_ = −0.180, *p* = 0.421, 95% CI [−0.559 0.261]) nor centro-parietal ROI’s (r_s_ = −0.395, *p* = 0.070, 95% CI [−0.700 −0.032]). We did not observe any further significant associations between SL-EC_D_, SL-EC_ND_, STEP_fw_ or STEP_bw_ and fronto-central or centro-parietal relative theta power.

## Discussion

With the present study we aimed to identify baseline neurophysiological markers associated with acute balance performance in healthy adults. For this purpose, resting-state EEG, short‐latency SEPs, and PPI were recorded prior to balance performance assessments on a slackline. Lower resting-state theta power correlated significantly with better SL-EO_ND_, whereas neither SEP amplitudes nor PPI measures related to any balance performance measure. These results highlight the potential of resting-state EEG spectral markers for rapid assessment of balance ability and suggest that SEP-based indices of long-term neuroplastic adaptation may not generalize to novices performing complex balance tasks.

The present findings support the proposition that spectral EEG power relates to individual differences in motor aptitude^31^. For example, in a digital-game paradigm, lower alpha and delta power forecasted superior subsequent motor performance^32^. Parietal theta power has been positively correlated with motor imagery accuracy, implicating theta synchronization in sensorimotor integration processes^33^. During isometric hand‐force control, alpha‐band fluctuations comodulate with reaction times, suggesting that alpha dynamics relate to preparedness for motor execution^34^. Moreover, enhanced right frontal and temporal power have been associated with improved skill acquisition in a ball‐rotation task, underscoring a region-specific relevance of EEG power measures in diverse motor performance contexts^35^. Although resting-state EEG power has been comparably underutilized in studies of motor performance, Lum et al. (2023) provide compelling evidence supporting its relevance, particularly with regard to theta power. In their study, participants who implicitly learned a visuospatial-motor sequence exhibited a significant negative correlation between resting-state relative theta power, measured at midline-frontal, right-frontal, and left-posterior sites, and sequence learning performance^36^. Critically, this relationship parallels the present finding that lower resting-state relative theta power correlates with superior acute slackline balance, suggesting a linkage between resting-state theta power and motor performance. To understand potential mechanisms underlying this relationship, it is imperative to consider the processes by which resting‐state theta oscillations may govern motor performance. Although resting‐state theta power has been more extensively examined in relation to cognitive than motor abilities^37^, its implications for neural efficiency may be comparable across domains. In children and adolescents, elevated resting-state theta power consistently correlates with poorer working memory performance^38^ and attentional deficits^39^. A similar inverse relationship has been observed between resting-state theta power and language proficiency^40^. Longitudinally, early individual differences in theta power have been shown to predict global cognitive outcomes into adulthood^37^, suggesting that baseline theta reflects enduring variations in neurocognitive function. Interestingly, during cognitive task performance, this pattern reverses, i.e., higher theta power is associated with improved performance^37^. A similar association is evident in the motor domain once our data are considered alongside a previous study. Investigating a potential link between cortical theta activity and continuous balance performance, Hulsdunker et al. (2015) found that higher theta power during task performance was associated with superior balance. Taken together, evidence from Hulsdunker et al. (2015) and the present study highlight a comparable disparity between the functional implications of theta power in resting-state conditions and during task performance. During rest, reduced theta power likely reflects a lower contribution of aperiodic, broadband activity. These irregular slow fluctuations can introduce neural noise and impair information processing, thereby denoting a more efficient baseline network state^37^. In contrast, increases in theta power during task engagement may represent narrowband synchronization within key networks that underpins active information encoding and sensorimotor integration^41^. This functional dichotomy could account for the seemingly paradoxical finding that both lower resting‐state theta and higher task‐related theta independently associate with superior motor performance, although further research is necessary to delineate these processes and their implications for motor abilities.

Although we observed a significant relationship between resting-state theta power and acute balance performance, we observed it only for the non-dominant leg. Establishing a clear metric for leg dominance is challenging^42^. In the present study, balance measures with eyes open and closed (SL-EO, SL-EC) did not differ between dominant and non-dominant limbs, indicating that dominant legs did not uniformly confer superior balance ability. Consequently, in the present study, some participants achieved better slackline performance with their non-dominant leg, while others did so with their dominant leg. This variability in limb-specific proficiency may have weakened the correlation for the dominant leg, resulting in statistically robust findings only for the non-dominant leg. Furthermore, no significant relationships between resting-state theta power and SL-EC or STEP were observed. Both STEP and SL-EC impose substantially higher sensorimotor and computational demands than SL-EO. The inclusion of rhythmic limb movements elevates central-nervous load^43^, and the removal of visual feedback dramatically increases reliance on proprioceptive and vestibular inputs^44^. On first exposure, these complexities may have compressed the spectrum of individual performance, i.e., participants clustered near floor levels of performance, thereby limiting variance and obscuring potential neurophysiological associations. Future work employing graduated task difficulty or practice paradigms may be necessary to reveal relationships across the full continuum of balance performance.

Contrary to expectations, neither SEP amplitudes nor paired-pulse inhibition (PPI) correlated with acute balance performance. This outcome can be ascribed to the considerable heterogeneity in reported associations between SEP amplitudes and motor ability. For example, athletes exhibited lower tibial P60 amplitudes^15^, as well as augmented N140 and P300 responses compared to non-athletes^45^. Furthermore, limb-specific increases in SEP amplitudes in football, racquet-sport, and baseball players were observed in previous research^16,18^. However, an equally robust set of studies has failed to detect any SEP amplitude differences between athletes and non-athletes^17,46–48^. These conflicting outcomes likely reflect substantial variability in methodology. Stimulation protocols, stimulation sites, intensities, and recording montages differ across protocols, making direct comparison challenging. Moreover, SEP paradigms have traditionally revealed chronic neuroplastic changes in elite athletic populations^14^. These paradigms may however lack the sensitivity to capture transient sensorimotor capacities in novices performing a novel balance task. In essence, SEP amplitude and related measures such as PPI may be more suited for the assessment of long-term exercise adaptations (e.g., skill learning) rather than the immediate, dynamic integration of multisensory inputs required for acute balance performance. Together, these considerations suggest that SEP and PPI markers show limited association with acute balance performance, at least in the present sample confronted with a highly challenging, unfamiliar slackline task.

Concerning limitations of the present study, some methodological considerations warrant acknowledgment. First, the custom EEG montage omitted the Fz electrode location to enable simultaneous SEP and EEG recordings across two systems, potentially reducing sensitivity to frontal theta activity. This limitation was addressed by employing region-of‐interest analyses across frontal and central electrode clusters, rather than relying on a single channel. Although analysis of relative power may obscure absolute power variations of interest, we chose to analyze relative rather than absolute spectral power to control for inter‐individual differences in skull thickness^49,50^. In addition, relative power analysis has demonstrated greater sensitivity to related performance associations in the cognitive domain^36,51,52^. In the present study, balance assessment centered on novel slackline tasks. While multiple conditions were included (walking, eyes-open, eyes-closed), comprehensive profiling of balance as a multifaceted motor skill would benefit from standardized test batteries. Furthermore, future studies would benefit from larger sample sizes. Beyond increasing statistical power, larger cohorts will be essential to capture the full spectrum of inter-individual variability in balance performance and neurophysiological markers, thereby improving the robustness and generalizability of findings across populations and task contexts. Another limitation concerns the assessment of leg dominance. In the present study, dominance was determined using a standardized self-report item from the Waterloo Footedness Questionnaire. While this provides a pragmatic and widely used measure, it does not capture the full complexity of dominance in tasks such as slacklining, where both legs contribute substantially to postural control. Future studies may therefore benefit from incorporating complementary, performance-based dominance assessments to provide a more nuanced perspective. Finally, our sample comprised healthy young adults naïve to slacklining, limiting generalizability. Including clinical and older populations in future research will enhance the broader applicability of our findings.

## Conclusion

Collectively, our findings underscore the promise of resting-state theta power for rapid, non-invasive assessment of specific balance performance. Future research should validate these results longitudinally, extend investigations to diverse balance paradigms and populations, and integrate multimodal neurophysiological measures to refine models of sensorimotor performance.

## Materials and methods

### Ethical statement

The study was endorsed by the local Ethics Committee of Leipzig University (Medical Faculty) (ref.no. 043/22-ek). All participants gave their written informed consent to participate in the study, in accordance with the Declaration of Helsinki.

### Participants

25 healthy participants (10 female) were enrolled in the present study (age: 24.0 ± 2.5, mean ± SD). Participants engaged in regular exercise for 3.6 ± 4.5 ^hrs^./_week_ over the past two years. None of the participants had any prior experience with slacklining. To assess individual footedness, we asked a single question of the Waterloo Footedness Questionnaire^53^ relating most to single-leg balance tasks (“*If you were to balance on one foot*,* which foot would you use*?”). Accordingly, 17 participants were right-leg dominant while 8 participants were left-leg dominant. When examining laterality-focused outcomes, we compared participants’ dominant versus non-dominant feet rather than simply right versus left.

Two participants had to be removed from overall analyses following outlier detection in behavioral parameters. Outliers were defined as values exceeding 1.5 times the interquartile range of the respective data. Due to faulty SEP measurements, 3 participants were additionally excluded. The remaining sample of 20 participants (7 female; age: 23.9 ± 2.5) was used for further SEP analyses. For EEG analyses, we removed 1 participant due to an erroneous measurement. The remaining sample of 22 participants (8 female; age: 23.8 ± 2.2) was used for further EEG analyses.

### Procedure

**Fig. 3:**
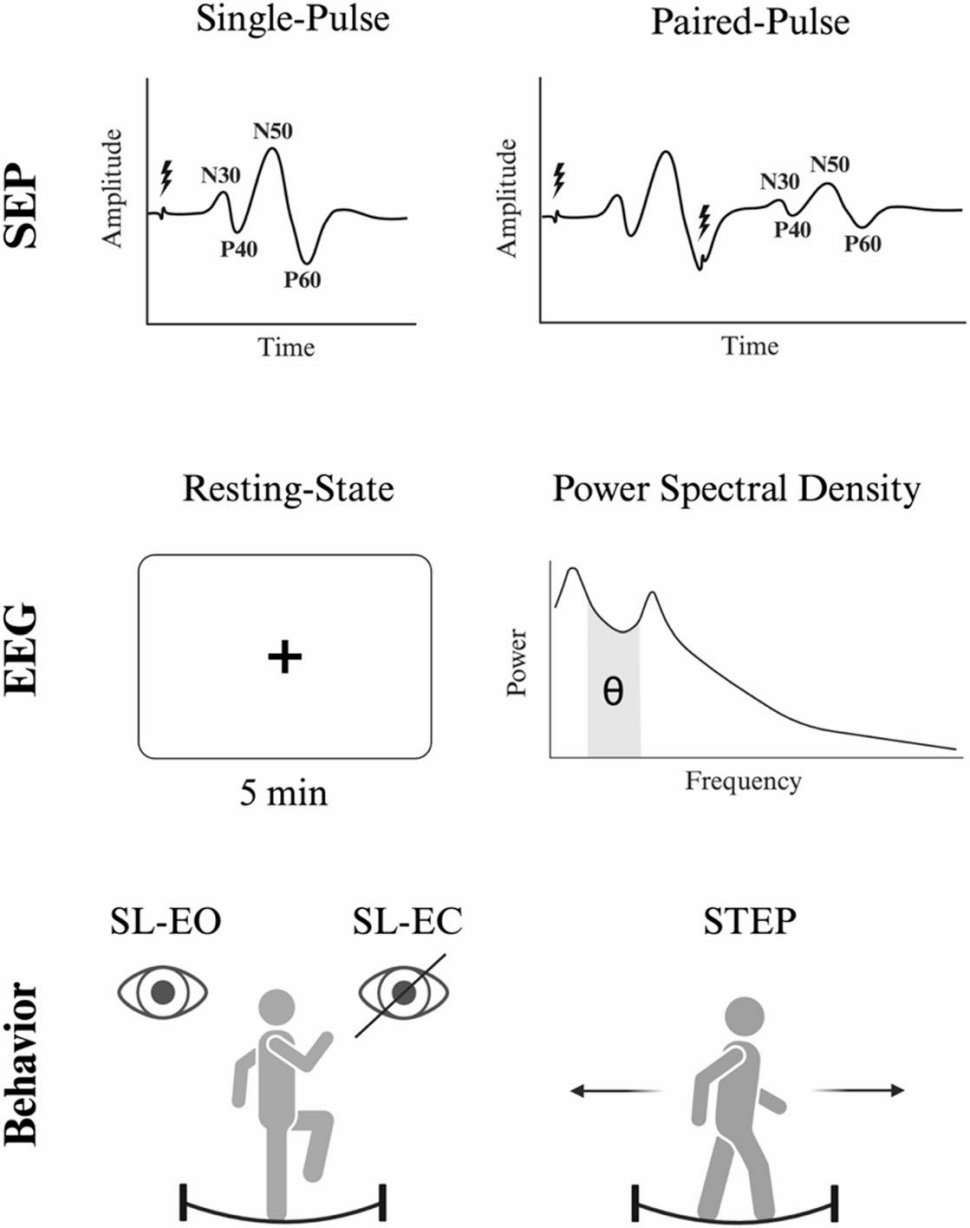
Study overview. Each row depicts one experimental modality: (1) SEP recordings using single (SP)- and paired-pulse (PP) stimulation, where black bolts mark stimulation onset times: 0 ms for single-pulse (SP) and both 0 ms and 60 ms for paired-pulse (PP) recordings; (2) resting-state EEG with power spectral density estimation in the theta band; and (3) slackline balance assessments: single-leg stance with eyes open (SL-EO), single-leg stance with eyes closed (SL-EC), and the successive-steps task (STEP) in forward and backward direction.

After collecting basic demographic information, the vertex of each participant was identified and served as a reference point for both SEP and EEG recordings. All participants first completed SEP measurements, followed by a 5 min. resting-state EEG and finally the behavioral experiment comprising three slackline-specific balance tasks (Fig. 3). All modalities are described in detail below.

#### Somatosensory-evoked potentials

Initially, each participant was fitted with a 32-channel EEG cap (Easycap GmbH, Germany) following the international 10–20 system, with the Cz electrode positioned at the vertex. The cap was modified by adding two 2.5 cm circular cut-outs: one at Fz and a second located 2 cm posterior to Cz (hereafter referred to as Cz’). This was done to enable the integration of both SEP and EEG systems. Here, we used the Nihon Kohden Neuropack X1 system (Nihon Kohden Corp., Japan) to record and analyze SEPs following tibial nerve stimulation. To improve impedance, areas around Fz and Cz’ were prepared using abrasive paste (OneStep AbrasivePlus Gel). Next, electrodes were affixed at Fz (reference electrode) and Cz’ (recording electrode) using conductive paste. Impedances were kept below 5kΩ. Participants then positioned themselves supine on a standard massage table. Tibial nerve stimulation was performed by applying a block electrode below the medial malleolus. SEP responses were sampled at 5120 Hz with an online bandpass filter set at 5–1500 Hz. To ensure adequate and reliable SEP signals, stimulation intensity was set at 2 mA above motor threshold, i.e., the minimum stimulation intensity to elicit a motor response in innervated muscles^20^. We conducted two stimulation types, single-pulse stimulation (SP) and paired-pulse stimulation (PP) per leg for each participant. For SP, 400 square-wave pulses (0.2 ms) were delivered at 3 Hz, while 400 paired square-wave pulses (0.2 ms; inter-stimulus interval: 60 ms) were delivered at 3 Hz during PP. The order of stimulation type, as well as the order of the stimulated leg was randomized across participants to avoid sequence effects. Averaged SEP traces per participant were used to assess latencies and peaks of N30, P40, N50, and P60. Peak-to-peak amplitudes between components N30-P40, P40-N50, and N50-P60 were also determined and used for statistical analyses. To estimate paired-pulse responses, we first subtracted single-pulse traces from paired-pulse traces, yielding a third trace. Paired-Pulse-Inhibition (PPI) was calculated for N30-P40, P40-N50, and N50-P60 as the peak-to-peak amplitude ratios of the third trace and the SP trace^20^. Notably, values of PPI greater than 1 indicate facilitation of synaptic transmission while values less than 1 indicate inhibition of synaptic transmission. All calculated PPI were used for subsequent statistical analyses.

#### EEG recordings

During resting-state EEG measurements, participants sat comfortably before a standard computer monitor and were instructed to fixate on a white cross displayed on a black background for five minutes. EEG data were recorded on a mobile 32-channel EEG system (LiveAmp, Brain Products GmbH, Germany) using an active electrode setup on the above mentioned, custom 32-channel EEG cap. Conductive gel (SuperVisc High-Viscosity electrolyte gel) was applied per electrode to ensure adequate impedance. Impedance of all electrodes was kept below 10 kΩ throughout the experiment. Data was transmitted wirelessly to a working station via a Bluetooth transmitter included in the LiveAmp module. EEG data were recorded with a sampling frequency of 500 Hz, an input impedance > 100 MΩ and a Common Mode Rejection Ratio (CMRR) > 80 dB. During recording, a band-pass filter between 1 and 100 Hz was used.

#### EEG preprocessing and spectral analysis

EEG preprocessing was carried out using the MATLAB-based eeglab toolbox^54^, as well as custom-written code in MATLAB (v. R2024a, The MathWorks Inc., Natick, USA). Data were band-pass filtered between 1 and 100 Hz and notch-filtered between 48 and 52 Hz to remove power line noise. Bad channels and noisy epochs were initially labeled using the clean_rawdata tool within eeglab and removed following manual inspection by a single trained researcher. Data were then re-referenced to the common average and subjected to independent component analysis (ICA) using the runica algorithm. Artifactual components were labeled using the ICAlabel tool within eeglab^55^. Final removal of artifactual components was carried out following conformity between components identified using ICAlabel and manual inspection of the identified artifactual components by a single trained researcher. EEG power was taken as power spectral densities of preprocessed resting-state EEG data, calculated using Welch’s method with a window size of 512 samples and 50% overlap^56^. Theta (4–8 Hz) power spectral density was estimated using the bandpower function in MATLAB and subsequently normalized to total power (across 1–100 Hz) to control for inter-individual differences. Relative theta power was subsequently averaged across specific channels to yield (1) a fronto-central region of interest (ROI; Fp1, Fp2, F3, F4, FC1, FC2) and (2) a centro-parietal ROI (Cz, CP1, CP2, C3, C4). The ROIs defined here closely align with those used in previous, related studies to improve comparability^28,57^. Finally, relative theta power within the fronto-central and centro-parietal ROIs were used for further statistical analyses.

#### Behavioral experiment

Following SEP and EEG measurements, participants performed three common slackline-specific balance tasks: Single-leg stance eyes-open (SL-EO), single-leg stance eyes-closed (SL-EC) and a successive-steps task (STEP)^58,59^. Single-leg stance tasks were performed on dominant (SL-EO_D_ & SL-EC_D_) and non-dominant legs (SL-EO_ND_ & SL-EC_ND_), while the stepping task was completed in forward (STEP_fw_) and backward (STEP_bw_) direction. Two slackline configurations were used during testing. The first featured a 4 m-long slackline mounted on a solid frame (Slackstar, Braun GmbH, Germany) set at a height of 55 cm, with small platforms positioned at each end to facilitate safe and comfortable mounting. This configuration was used for SL-EO and STEP. A second configuration featured the GIBOARD (Gibbon, ID Sports GmbH, Germany), a 1 m-long mounted slackline set at a height of 14 cm. This configuration was used for SL-EC to reduce risk of injury during the eyes-closed condition. In each condition, participants completed exactly two trials and the higher score was used for analysis. This approach mitigates the influence of unrepresentative, accidental failures (e.g., an immediate fall) on our outcome measures, while constraining exposure to the task, preserving the assessment of true, initial performance in our task-naïve cohort by minimizing familiarization effects^59^. For the SL-EO and SL-EC tests, participants stood on one leg in the middle of the slackline while the score was the maximum duration that participants maintained a single-leg stance without ground contact. For the STEP test, the score was the total number of consecutive successful steps taken forward and backward between platforms^58^. In case of a complete crossing, participants immediately began walking in the opposite direction, with all steps being counted. Participants completed all tasks in succession separated by 15 s breaks in between trials. Throughout all slackline tasks, two researchers stood nearby to prevent injury in the event of a fall. Again, task order and starting legs were randomized to avoid sequence effects.

#### Statistical analysis

Statistical analyses were carried out using JASP (version 0.19.3; University of Amsterdam, Amsterdam, Netherlands). Initially, normal distribution of all relevant variables was assessed using Shapiro-wilk tests (α = 0.05). The majority of SEP amplitudes, latencies, and stimulation intensities, as well as relative theta PSDs were normally distributed, while the majority of behavioral outcome measures were non-normally distributed. Consequently, comparisons of balance performance between dominant and non-dominant legs were realized using Wilcoxon signed-rank tests, while differences in stimulation intensities between dominant and non-dominant legs were compared using paired t-tests. To test PPI, a two-sided one-sample t-test (reference value: 1) was conducted per component pair. Differences in SEP amplitudes and PPI between the dominant and non-dominant leg were assessed via a repeated-measures ANOVA with the within-subject factors *Component* (N30–P40, P40–N50, N50–P60) and *Leg* (dominant vs. non-dominant). Lastly, to explore potential associations between SEP amplitudes and/or relative theta power and initial balance performance, Spearman rank correlation analyses were performed. Correlation analyses were conducted at two levels: (A) EEG x Performance and (B) SEP x Performance. Within each level, separate analyses were performed for each balance outcome (SL-EO, SL-EC, STEP-F, STEP-B). For each ROI (fronto-central and centro-parietal), theta power was correlated with the performance measures. Bonferroni correction was applied across the two ROIs, resulting in a corrected threshold of α = 0.025. For each stimulation condition (SP and PP) and leg (dominant and non-dominant), correlations were performed separately for the three SEP amplitude components (N30-P40, P40-N50, N50-P60). Bonferroni correction was therefore applied across these three components, yielding a corrected threshold of α = 0.017.

## Data Availability

Data availability statement: The original data presented in the study are openly available at: https://figshare.com/articles/dataset/Data_-_Slacklearn/28903037.
